# In-silico investigation into optimal photoacoustic probe design using a digital thyroid phantom

**DOI:** 10.1117/1.JBO.32.S1.S14902

**Published:** 2026-09-09

**Authors:** Max T. Rietberg, Jeroen Veltman, Jelmer M. Wolterink, Srirang Manohar

**Affiliations:** a University of Twente, TechMed Centre, Multi-Modality Medical Imaging, Enschede, The Netherlands; b Hospital Ziekenhuisgroep Twente, Radiology Department, Hengelo, The Netherlands; c University of Twente, TechMed Centre, Department of Applied Mathematics, Enschede, The Netherlands

**Keywords:** thyroid, nodule, photoacoustic imaging, phantom, in silico

## Abstract

**Significance:**

Thyroid nodules have a high incidence, but a low malignancy rate, placing demands on imaging specificity. Photoacoustic imaging is being studied as a method that could potentially help increase the unsatisfactory specificity of ultrasound, the current first-line imaging method, which would decrease the number of unnecessary clinical interventions.

**Aim:**

We employ a digital thyroid phantom and numerical simulations to evaluate photoacoustic probe designs on their suitability for thyroid nodule risk estimation.

**Approach:**

Using the SIMPA toolkit, we examined 1080 probe configurations with varying illumination geometry, detector shape, pitch, and wavelength combinations. The performance of each probe was quantified on its sensitivity and its accuracy of oxygen saturation estimation.

**Results:**

Optimal performance was found using wavelengths 757 and 800 nm, and a curved detector (0.5 split, 0.40 mm pitch) for external illumination. However, performance increases significantly with endotracheal illumination, using wavelengths of 700 and 800 nm, and a curved detector (0.25 split, 0.40 mm pitch).

**Conclusions:**

These results could provide design guidance for optimizing photoacoustic hardware, assisting with the clinical adoption of PAI, which could improve the diagnosis of thyroid nodules.

## 1 Introduction

Thyroid nodules are highly prevalent, with an estimated incidence of up to 65% in all adults.^1^ Although the vast majority of nodules (up to 96%^1^) are benign, nearly 600,000 new cases of thyroid cancer are diagnosed worldwide every year.^2^ The current first-line imaging modality for thyroid nodule risk assessment is ultrasound (US), in which nodules are stratified using feature-based systems. The most commonly used in clinical practice is the Thyroid Imaging Reporting and Data System (TI-RADS), which categorizes nodules into five risk levels, ranging from benign to highly suspicious.^3^ However, although TI-RADS has high sensitivity, its specificity is limited. For example, the American College of Radiology (ACR) version of the TI-RADS has a reported specificity of only 52.9%.^4^ This low specificity leads to a substantial number of indeterminate findings. İlhan Hekimsoy et al.^5^ reported that 44% of nodules were classified as ACR TI-RADS 3 (i.e., mildly suspicious), even though only 13% of nodules in this category were malignant. Ambiguous and suspicious cases are typically followed up or evaluated with US-guided fine-needle aspiration (FNA), yet ∼70% of FNAs yield benign diagnoses.^6^ In cases where FNA results remain inconclusive, hemithyroidectomy is often recommended, despite up to 80% of these surgeries ultimately revealing benign pathology.^7^ These unnecessary clinical interventions place a significant burden on patients, healthcare systems, and, therefore, society, underlining the urgent need for improved first-line, noninvasive imaging methods with greater specificity for thyroid nodule risk stratification.

Photoacoustic (PA) imaging (PAI) is a hybrid imaging method that exploits optical absorption–induced thermoelastic expansion to generate ultrasound signals for imaging.^8^ In PAI, the tissue is illuminated with a pulsed laser. Various naturally occurring chromophores absorb transmitted photons, and the absorbed energy is translated into heat and a corresponding rise in local pressure. Next, this pressure will propagate throughout the tissue as sound waves, which ultrasound transducers can detect. These recorded signals can then be used to reconstruct the PA image using inverse methods, such as backprojection or time-reversal algorithms.^9^ PAI delivers functional and molecular information from tissue, e.g., vasculature maps and oxygen saturation (${\mathrm{SO}}_{2}$).^9^ These biomarkers might be useful for risk stratification of tumour tissue, as they can differ between benign and malignant tissue.^10^ For instance, Roll et al.^11^ and Tang et al.^12^ found that malignant thyroid nodules had significantly lower ${\mathrm{SO}}_{2}$. Adding these features to the TI-RADS system could decrease false positives and unnecessary medical procedures, reduce patient stress, and lower healthcare costs.^13^ Kim et al.^14^ and Ahn et al.^15^ proposed improvements for the American Thyroid Association guidelines by extending them to include various PAI-derived parameters: the PA spectral gradient, mean ${\mathrm{SO}}_{2}$, and the skewness of the ${\mathrm{SO}}_{2}$ distribution. However, as PAI is relatively new, neither hardware (e.g., optical wavelengths and detector shape) nor software has been optimized for thyroid nodule assessment, and imaging might be affected by artifacts. Due to PAI’s dual-physical nature, artifacts in PAI are diverse.^8^ For the specific setup of handheld (curvi)linear thyroid PAI, the most dominant artifacts include (1) limited view (which we hypothesize to be the cause of the ripple-like distortions in Fig. 2(c) of Roll et al.^11^); (2) fluence decay and spectral colouring, e.g., due to strong water absorption;^13^ (3) reflection artifacts, from e.g., probe^15^ or multiple interfaces in the subcutaneous fat;^13^ and (4) limited frequency response. Artifacts can be mitigated to some degree; the spectral colouring artifact can be reduced with suitable fluence-compensation algorithms, for example, and can also be less pronounced when using illumination wavelengths that are more closely spaced together.^8^ Furthermore, limited-view artifacts decrease as the detection surface and thus the probe size increase, which must be balanced with ease of use in a handheld scanner.^8^ However, the intensity and number of artifacts (and thus image quality) cannot be predicted without dedicated physical or digital experiments, in carefully controlled situations.

Regulations demand that clinical studies and evaluations be performed before any imaging device can be used for patient care. However, studies on patients are expensive in terms of both time and cost. Another, less expensive option is studies with physical phantoms that mimic the patient in shape and physical properties. However, both studies with patients and with physical phantoms lack a known ground truth (e.g., the initial pressure or the exact physical properties). Moreover, physical phantoms can only approach the physical properties seen in patients. In PAI, the International Photoacoustic Standardisation Consortium (IPASC) formulated phantom design criteria to enhance comparison of preclinical PA measurements.^16^ Studies with digital phantoms offer a compelling alternative to physical phantoms. Digital phantoms are computational models that numerically represent the anatomical structures and physical properties relevant to the imaging modality under investigation. Using such models, forward simulations using a digital twin of the imaging system can be performed that describe the propagation of the relevant form of energy (e.g., light and ultrasound waves in PAI) from source to detector under controlled conditions. Digital phantoms offer high realism, known ground truth, and low cost, enabling fast testing. Therefore, studies with digital phantoms are ideal for the first stage of the development or testing process for a medical device.^17^ The development of anthropomorphic, digital PAI phantoms is an ongoing research, e.g., a series of breast phantoms^18^ and a series of prostate phantoms.^19^

Despite promising clinical results, the influence of hardware design parameters on PA thyroid imaging performance has not been systematically studied. In this paper, we investigate the use of PAI hardware for thyroid nodule imaging by evaluating the performance of a large number of distinct hardware configurations on a digital thyroid phantom. First, we define a digital thyroid phantom and a digital twin of an existing PAI system. Next, we evaluate the impact of the illumination location, detector split, detector shape, pitch, and optical wavelengths of the digital twin. Our findings may help to accelerate the clinical adoption of PAI, by providing insight into optimizing PAI systems for thyroid nodule imaging, which we formulate into concrete design recommendations.

## 2 Methods

In this work, we (1) define the simulation volume and the digital phantom, by defining the anatomical structures geometrically and assigning specific physical parameters; (2) define the digital twin (i.e., light sources and ultrasound detectors); (3) generate simulated measurements (i.e., the digital phantom studies); and (4) analyze the simulated measurements.

### 2.1 Definition of the Simulation Volume and the Digital Phantom

**Fig. 1:**
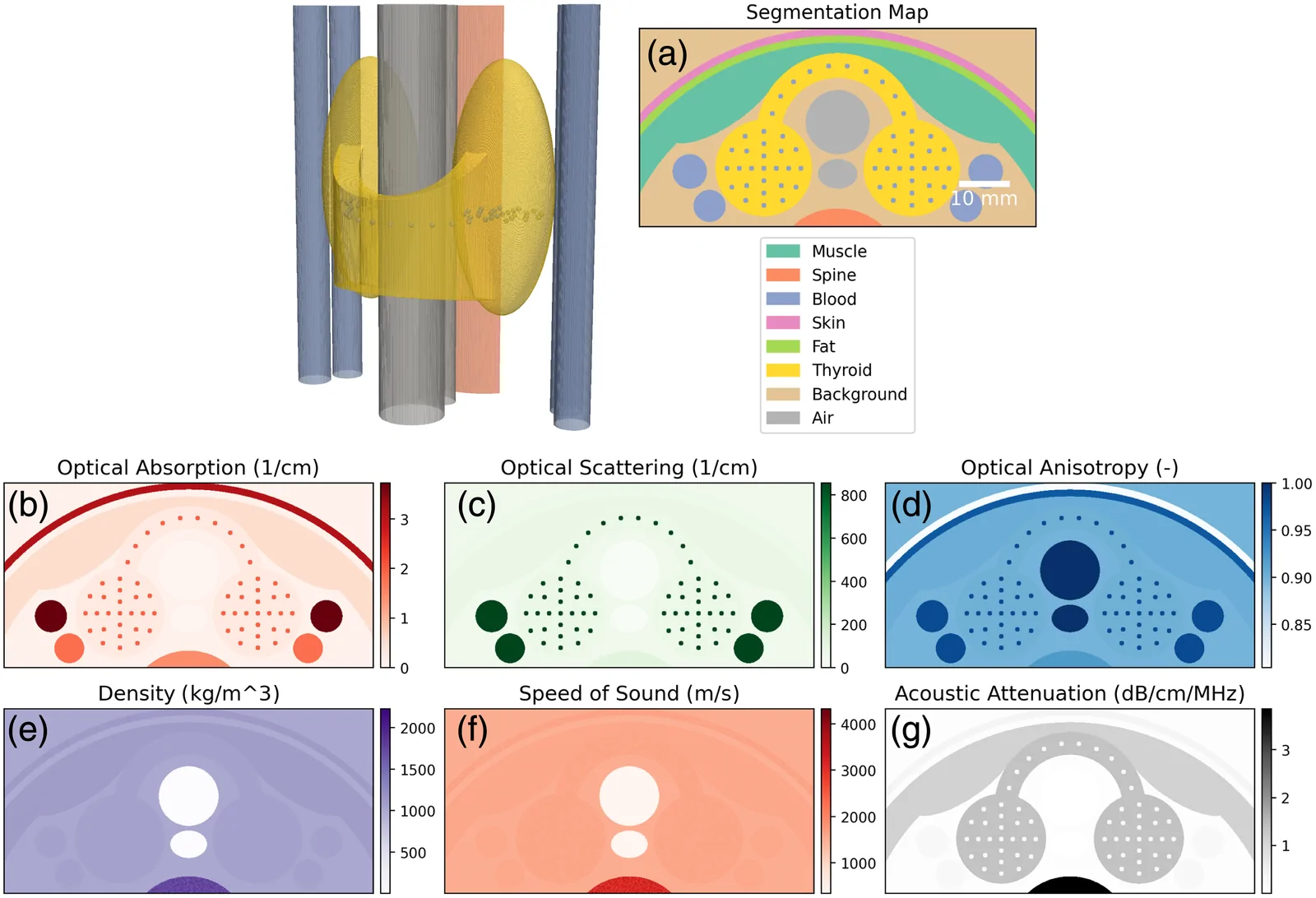
Visualization of the phantom in 3D, and as a transverse slice (top), and visualizations of its optical properties (for an illumination of 757 nm) and acoustic properties (bottom).

The size of the simulation volume is set to 80.4×80.4×40.2  mm, wherein the digital phantom is created by combining relevant anatomical structures. The phantom shape, together with its physical properties, is shown in Fig. 1. First, the neck is defined as a cylinder with a radius of 5.5 cm, based on an average circumference of 34.7 cm.^20^ We define three shell layers inside the cylinder, each with a thickness of 0.15 cm: skin (epidermis with a melanin volume fraction of 0.014), fat (with the physical properties of subcutaneous fat), and muscle (with an ${\mathrm{SO}}_{2}$ of 0.175), from anterior to posterior. The muscle layer is thickened at several places to emulate the effect of the sternocleidomastoid and surrounding muscles. Next, the common carotid artery and internal jugular vein are modeled as cylinders, with a radius and ${\mathrm{SO}}_{2}$ of 0.325 cm and 0.97, and of 0.35 cm and 0.73, respectively. The trachea is modelled as a cylinder with a radius of 0.65 cm, filled with air; the oesophagus as a elliptic cylinder with a semi-minor and semi-major axes of length 0.4 and 0.95 cm, respectively, also filled with air; and the spine as a cylinder with a radius of 1.35 cm, made of the bone. The thyroid itself is based on the phantom as designed by Hermosilla et al.,^21^ see Appendix A. When required (see Sec. 2.4), we add targets that model (a part of) the nodule as a 0.5-mm-radius sphere filled with blood. These 60 targets are placed systematically throughout the thyroid, all in the imaging plane. Finally, all voxels that are not part of a structure are filled with generic soft tissue.

The majority of the physical properties can be automatically set using the libraries in the Simulation and Image Processing for Photonics and Acoustics (SIMPA) toolkit,^22^ an open-source toolkit that simulates optical and acoustic imaging modalities. However, thyroid tissue is not included in SIMPA, which is why we use the optical properties from Dubal et al.,^23^ speed of sound from Miura and Mineta,^24^ and the remaining acoustic properties from the IT’IS tissue database.^25^ Furthermore, the optical values in SIMPA are extended beyond the default 1000 nm limit. Namely, the absorption coefficient of fat,^26^ water,^27^ and oxyhemoglobin and deoxyhemoglobin^28^ are extended using literature-based values. The other values (e.g., the absorption coefficient of melanin) are extended by fitting and extrapolating a second-degree polynomial using the existing log10 values. Next, to enhance the acoustic realism, instead of having a region-wise consistent speed of sound and density (i.e., inside a region belonging to a specific tissue type, the values are spatially homogeneous), we assign values per voxel according to normal Gaussian distributions, with a standard deviation according to Miura and Mineta^24^ and the IT’IS tissue database.^25^ For all tissue types, the Grüneisen parameter is calculated using the empirical equation (0.0043+0.0053×T), where T is the temperature in Celsius,^29^ which is fixed at 37°C, as is default in SIMPA.

### 2.2 Definition of the Digital Twin

We use the probe characteristics of the Imagio system (model 9005, Seno Medical Instruments, Inc, San Antonio, Texas)^30^ to develop a base digital twin that serves as the reference configuration for hardware modifications. This combined US and PA imaging system is FDA-approved and was originally developed for breast imaging. The probe comprises a conventional handheld linear ultrasound array, integrated with dual-sided optical illumination for PA excitation. The illumination consists of two rectangular beams of 6×43  mm, both using wavelengths of 757 and 1064 nm, and the ultrasound detection consists of 128 elements arranged in a linear fashion sandwiched between the illumination beams, with a pitch of 0.30 mm and an acoustic bandwidth of 9.4  MHz±80%. A digital twin of this probe is made by replicating its behavior with SIMPA.^22^

**Fig. 2:**
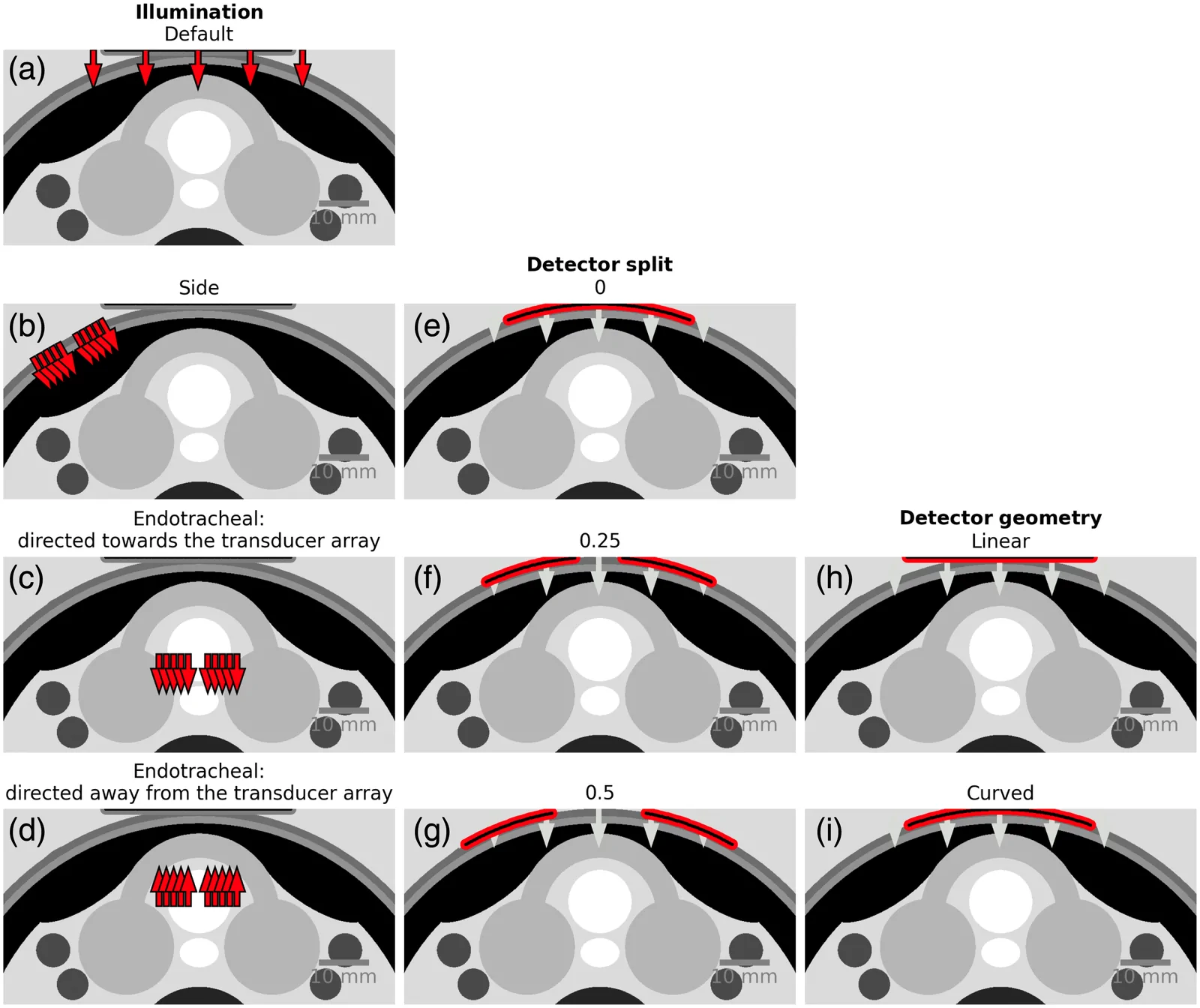
Visualization of probe configurations with varying (a)–(d) illumination location, with illumination shown in red; (e)–(g) detector split, with acoustic elements shown in red; (h) and (i) detector shape options, with acoustic elements shown in red.

With the starting point modeled, we modify its properties to investigate their effect on the probe’s performance. Figure 2 and Table 1 show the parameters that are changed from the original design, specifically: illumination location, detector split, detector shape, pitch, and optical wavelengths. The optical wavelengths are set between 700 and 1000 nm, in 100 nm increments, as well as the two default wavelengths of the Imagio system, i.e., 757 and 1064 nm. For the wavelengths, we explore all combinations of two wavelengths (e.g., 700 and 757 nm, 700 and 800 nm, etc).

**Table 1:** Design properties of the probe and their explored options.

Design parameter	Setting
Illumination location	Default, side, endotracheal (directed toward the detector array), and endotracheal (directed away from the detector array)
Detector split	0, 0.25, and 0.5
Detector shape	Linear and curved
Pitch	0.30, 0.35, and 0.40 mm
Optical wavelengths	700, 757, 800, 900, 1000, and 1064 nm, choose two

To test the effect of the illumination placement, we examine not only the default setting (i.e., illumination beams coupled to the transducer) but also cases where the illumination is detached and placed laterally to the side of the neck and cases where the illumination is detached and placed endotracheally, see Figs. 2(a)–2(d). Furthermore, we examine the effect of a detector split [see Figs. 2(e)–2(g)] and detector shape [linear or curved, see Figs. 2(h)–2(i)]. The detector split is defined as the relative space between the left and right halves of the detector array. If a probe with 0 split would be 10 cm wide, then one with a 0.5 split would be 15 cm wide: two detector arrays with a width of 5 cm with 5 cm of empty space (0.5×5  cm) in the middle.

### 2.3 Generating Simulated Measurements

We explore a large number of designs. This is done by performing a grid search, evaluating every combination of the design parameters (see Table 1). This results in (62)×4×2×3×3=1080 combinations. To reduce computational burden, the evaluation strategy is split into two steps: a full grid search with two key simplifications, followed by a targeted (but more accurate) evaluation of the top 30 setups. The top 30 setups of the first step are determined by sorting all setups on their combined metrics, see Sec. 2.4. The simplifications during the first step are (1) combining the two illumination bars into one bar of 16×43  mm and (2) decreasing the acoustic forward resolution to 0.2 mm (see below). In the second step, this is not the case; the two illumination bars are kept split, and the acoustic resolution is kept at 0.1 mm.

The simulations are performed using SIMPA,^22^ which facilitates realistic PAI digital measurements using MCX^31^ and k-Wave.^32^ For all our simulations, we use a volume of ∼40×80×80  mm, with an isotropic resolution of 0.1 mm for the MCX simulations and the reconstruction. The acoustic forward simulations in k-Wave are performed after, for the first step (wide, simplified grid search), downscaling to a resolution of 0.3 mm and then upscaling to a resolution of 0.2 mm, and for the second step (specific but more realistic evaluation), downscaling to a resolution of 0.2 mm and then upscaling to a resolution of 0.1 mm. This achieves two goals: decreasing computational time (for the first step) and preventing the formation of Fourier artifacts (due to hard edges, for both steps). Interpolation is performed using the trilinear RegularGridInterpolator from SciPy.

In the SIMPA pipeline, first, the phantom is constructed, and its spatially varying physical properties are assigned. Next, the optical forward simulation, down- and upscaling, and acoustic forward simulation are performed in order. Zero-mean Gaussian noise (which is often used in literature^18^^,^^33^^,^^34^) with a standard deviation of $10^{-3}\;\mathrm{Pa}$ is added to the resultant time series. The noisy time series are deconvolved with the known frequency response, and finally, the reconstruction is performed, which is an iterative 2D time-reversal technique with an assumed speed of sound of 1540  m/s and a maximum of 5 iterations. After reconstruction, the Fast Linear Unmixing for PhotoAcoustic Imaging (FLUPAI) technique from SIMPA is used to determine ${\mathrm{SO}}_{2}$ values.

### 2.4 Analysis of the Simulated Measurements

The evaluation of a design consists of running four simulations: one without targets, one with 60 targets with an ${\mathrm{SO}}_{2}$ of 0.75, one with 60 targets with an ${\mathrm{SO}}_{2}$ of 0.85, and one with 60 targets with an ${\mathrm{SO}}_{2}$ of 0.95. These simulations are combined to calculate the impact of target presence on the reconstructed pressure $p_{\mathrm{recon}}$ (as a proxy for sensitivity) and the accuracy in ${\mathrm{SO}}_{2}$ estimation (since this ${\mathrm{SO}}_{2}$ is a PAI biomarker that has proven its utility^15^), which together determine its performance score. The mean maximum increase (MMI) is calculated by

$$p_{\mathrm{recon},\text{increase}}(x,y;\lambda ,{\mathrm{SO}}_{2})=p_{\mathrm{recon}}^{\text{target}\text{ at}(x,y)}(x,y;\lambda ,{\mathrm{SO}}_{2})-p_{\mathrm{recon}}^{\text{no}\;\mathrm{target}}(x,y;\lambda ),$$ (1)

$$\mathrm{MMI}=\frac{1}{3}\overset{0.75,0.85,0.95}{\underset{{\mathrm{SO}}_{2}}{\sum }}\frac{1}{N}\overset{N}{\underset{i=1}{\sum }}\underset{\lambda \in \lambda_{1},\lambda_{2}}{\max}(p_{\mathrm{recon},\text{increase}}(x_{i},y_{i};\lambda ,{\mathrm{SO}}_{2})),$$ (2)

where N is the number of targets (which is set to 60), $x_{i}$ and $y_{i}$ are the coordinates of the target i, and $\lambda_{1}$ and $\lambda_{2}$ are the wavelengths of the setup. The mean squared error in ${\mathrm{SO}}_{2}$ estimation (${\mathrm{MSE}}_{{\mathrm{SO}}_{2}}$) is calculated by

SE(x,y;SO2)=(SO2(x,y)−SO^2(x,y))2, (3)

$${\mathrm{MSE}}_{{\mathrm{SO}}_{2}}=\frac{1}{3}\overset{0.75,0.85,0.95}{\underset{{\mathrm{SO}}_{2}}{\sum }}\frac{1}{N}\overset{N}{\underset{i=1}{\sum }}\mathrm{SE}(x_{i},y_{i};{\mathrm{SO}}_{2}),$$ (4)

where ${\hat{\mathrm{SO}}}_{2}$ is the estimated ${\mathrm{SO}}_{2}$, given the setup. Previous research has found only a small difference in nodule incidence between the left and right lobes and that a minority of nodules occur in the isthmus (6% to 9%), which has a smaller volume, however.^35^^,^^36^ Therefore, we will not attach specific weightings based on nodule location.

## 3 Results

### 3.1 Demonstration Using the Default Hardware

**Fig. 3:**
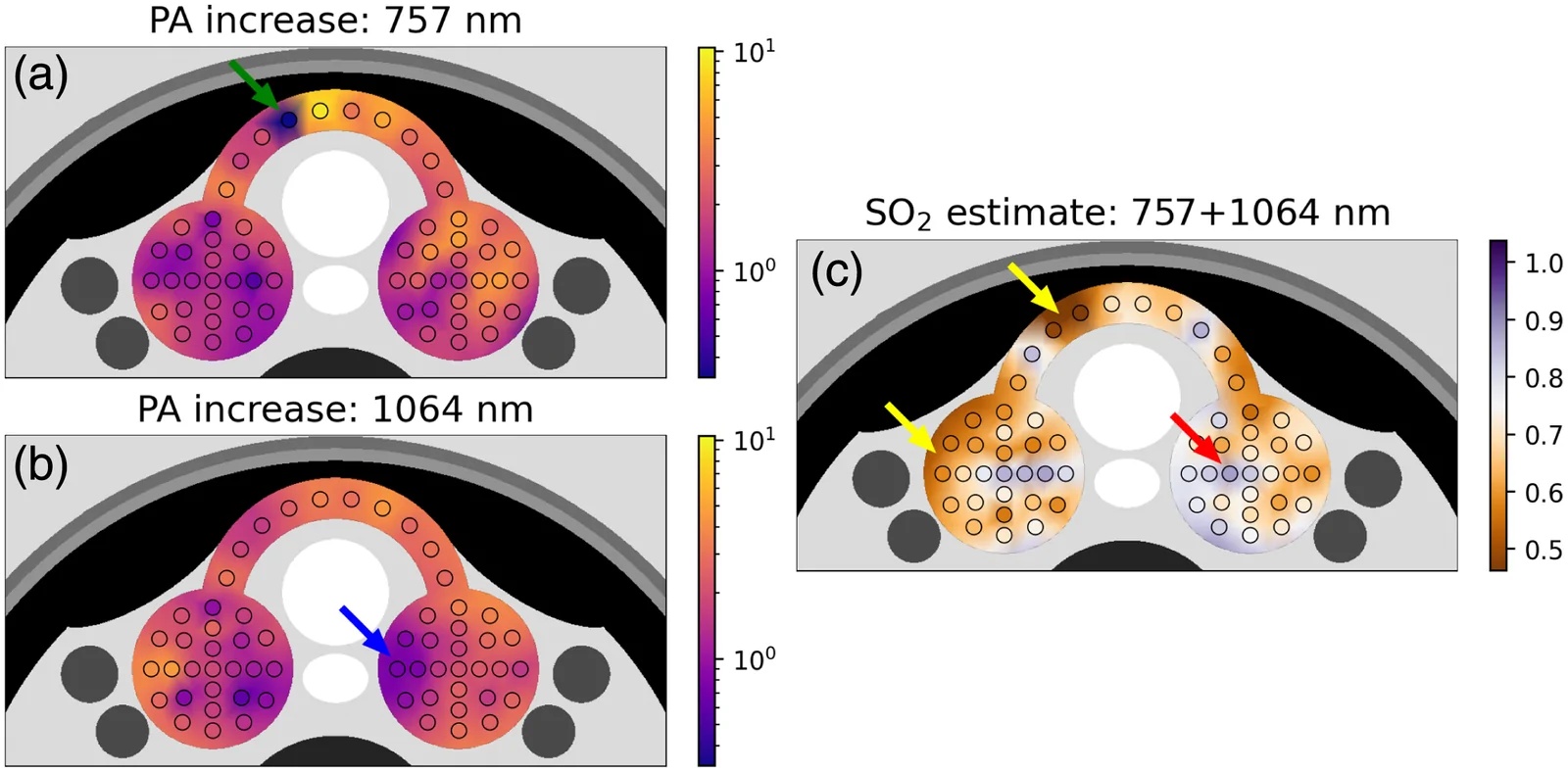
Demonstration of PAI simulation on the phantom with default hardware, at 757 and 1064 nm. (a), (b) PA amplitude increase for different target locations. (c) ${\mathrm{SO}}_{2}$ estimate (ground truth is 0.75) for different target locations (right). The values in between the targets are interpolated using Gouraud shading. The green arrow in panel (a) indicates a target that resulted in a lower PA increase due to noise. The blue arrow in panel (b) indicates several targets that showed lower PA increases due to acoustic reflections from the trachea and limited viewing. The yellow arrows in panel (c) indicate various targets which have an underestimated ${\mathrm{SO}}_{2}$, and the red arrows in panel (c) indicate some targets which have an overestimated ${\mathrm{SO}}_{2}$, due to noise, limited view, and/or limited frequency response.

As a baseline, we first evaluate the default hardware (i.e., the unchanged Imagio system). The PA amplitude increase as defined in Eq. (1) for the default hardware configuration at 757 and 1064 nm is shown in Figs. 3(a) and 3(b), respectively, and the estimated ${\mathrm{SO}}_{2}$ using the FLUPAI technique in Fig. 3(c). Several types of artifacts degrade the probe’s performance, with fluence decay and limited view [e.g., see blue arrow in Fig. 3(b)] being the most pronounced, but reflection artifacts and limited frequency response are also influential. The limited view artifacts in the underlying PA images match the ripple-like distortions in Fig. 2(c) of Roll et al.,^11^ and the reflection artifacts from the probe were also visible like the ones discussed in Ahn et al.^15^ However, the reflection artifacts due to multiple interfaces in the subcutaneous fat as shown in Noltes et al.^13^ were not visible in our measurements, as our phantom did not include these specific patterns in the fat. Fluence decay is one of the major causes of a depth-dependent decrease in PA intensity^8^ and is therefore present throughout our images. Namely, the increase in PA intensity decreases with depth, partly owing to fluence decay. Furthermore, the metrics are not symmetric; i.e., the left and right lobes show different patterns due to the added noise in the time series [see green arrow in Fig. 3(a)]. Artifacts that affect the PA increase often indirectly affect ${\mathrm{SO}}_{2}$ estimation, which explains why distortions in ${\mathrm{SO}}_{2}$ are regularly visible in the PA increase at one or both wavelengths, e.g., see yellow and red arrows in Fig. 3(c) for under- and overestimated ${\mathrm{SO}}_{2}$, respectively.

### 3.2 Investigation of Design Parameters

#### 3.2.1 First step: grid search

**Fig. 4:**
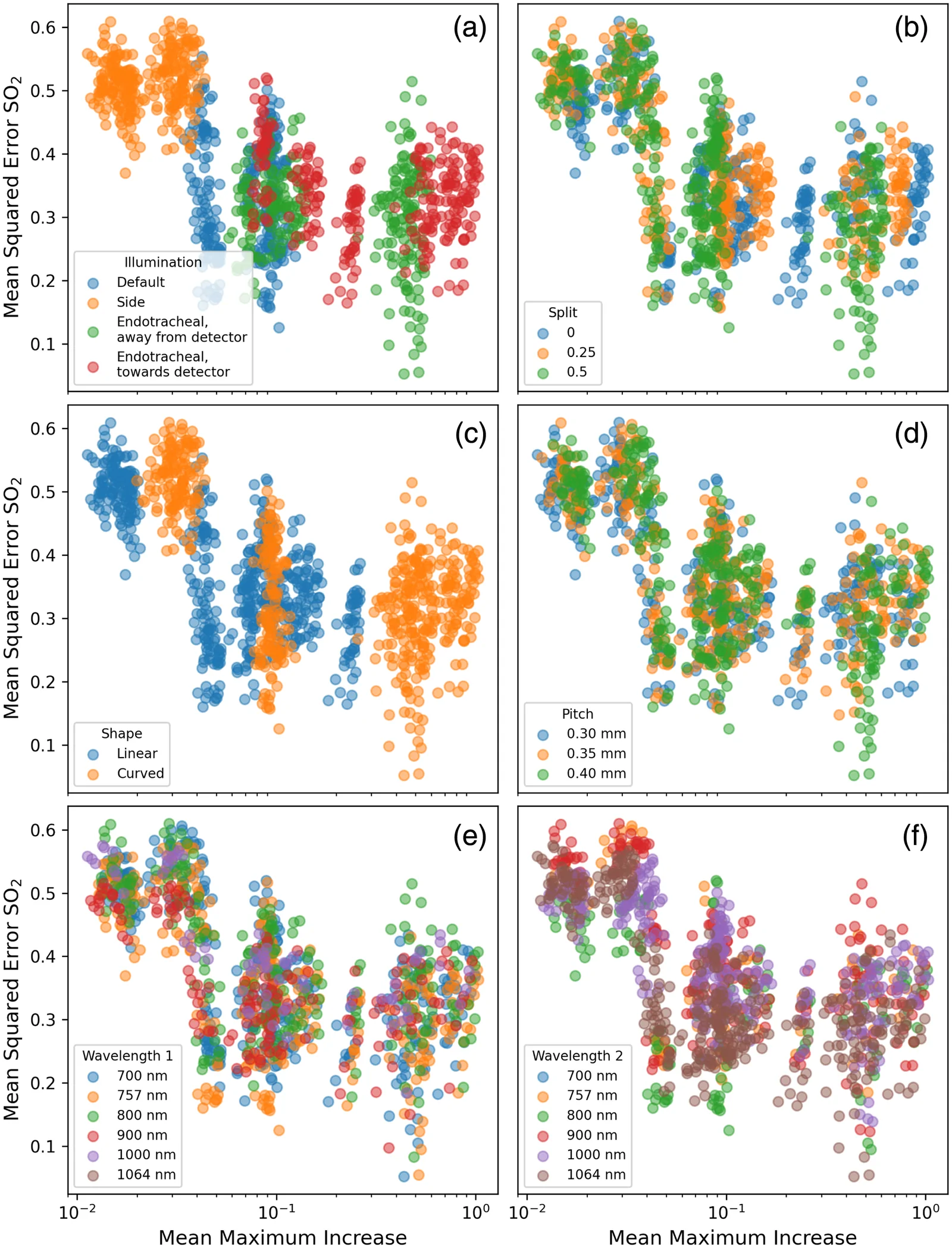
Evaluation of all 1080 hardware combinations on MMI and ${\mathrm{MSE}}_{{\mathrm{SO}}_{2}}$. We investigate six design parameters. All subfigures show the same results, but with a different design parameter highlighted. (a) Illumination location, (b) detector split, (c) detector shape, (d) pitch; (e) and (f) optical wavelengths.

As mentioned, the first step is a grid search, in which we evaluate every combination of the design parameters on MMI and ${\mathrm{MSE}}_{{\mathrm{SO}}_{2}}$ (see Table 1). The metrics for all combinations are shown in Fig. 4. To ease interpretation, we present the results in six subfigures, each highlighting one design parameter: illumination location, detector split, detector shape, pitch, and optical wavelengths. Probes with similar illumination locations are clustered [see Fig. 4(a)], indicating that the illumination location is one of the most influential design parameters. Although the two endotracheal options score best, especially for MMI, side illumination scores the worst on both metrics. The latter can be attributed to the increase in the distance between the illumination and the targets, leading to greater fluence decay for side illumination compared with other locations. Furthermore, given an illumination location, the two detector shapes differ strongly in their scores [see Fig. 4(c)], with the curved detector option generally correlating with increased MMI. The other design parameters are harder to interpret visually, but the wavelength combination of 757 and 800 nm for the default illumination, and the wavelength of 1064 nm for endotracheal illumination, are noticeable for their low ${\mathrm{MSE}}_{{\mathrm{SO}}_{2}}$ [see Figs. 4(e) and 4(f)].

Analyzing the correlation between design parameters and MMI using a full-factorial ANOVA reveals that performance variance is almost entirely defined by the interaction between illumination location and detector shape (see Appendix B) and to a lesser extent by the interaction between illumination and array split. By contrast, first-order (main) effects of all parameters are negligible when averaged across the design space, indicating that these parameters cannot be optimized independently. We observe that endotracheal illumination and a curved detector array both lead to higher MMI, with directing the illumination toward the detector array, and thus away from the majority of the targets, performing best. Given endotracheal illumination, a curved detector with no split and pitch of 0.40 mm maximizes the MMI.

Next, the ANOVA on ${\mathrm{MSE}}_{{\mathrm{SO}}_{2}}$ reveals that a large number of interactions dominate the error structure (see Appendix B). This contrasts with the MMI analysis, in which a single effect could explain a large majority of the results. We observe that endotracheal illumination leads to lower ${\mathrm{MSE}}_{{\mathrm{SO}}_{2}}$ and that illumination directed away from the detector array, and thus toward the majority of the targets, performs best. Given the default illumination location, the wavelength combinations of 757 and 800, 700 and 800, and 700 and 757 nm minimize the ${\mathrm{MSE}}_{{\mathrm{SO}}_{2}}$, in decreasing order. However, given side illumination with 757 and 800, 900 and 1000, and 700 and 800 nm, minimize the ${\mathrm{MSE}}_{{\mathrm{SO}}_{2}}$; and given one of the two types of endotracheal illumination, 700 and 1064, 757 and 1064, and 800 and 1064 nm, minimize ${\mathrm{MSE}}_{{\mathrm{SO}}_{2}}$. Although the illumination location determines how much and what type of tissue the light will encounter before reaching the targets, the wavelength combinations determine the amount and type of spectral colouring that occurs based on the given tissue.

#### 3.2.2 Second step: specific evaluation

The second step consists of a more specific (but more accurate) evaluation of the top 30 setups from the first step, determined by sorting all setups by their log 10 MMI z-score (which should be high, see Sec. 2.4) minus their ${\mathrm{MSE}}_{{\mathrm{SO}}_{2}}$ z-score (which should be low, see Sec. 2.4). These setups include the two endotracheal illumination locations, only the curved shape, all three split and pitch options, and various wavelength combinations. The results of the evaluation are shown in Appendix C, sorted on their log 10 MMI z-score minus their ${\mathrm{MSE}}_{{\mathrm{SO}}_{2}}$ z-score. We observe that the MMI of these 30 setups increased from 0.56±0.15 in the first step to 3.88±0.30 in the second step, and the ${\mathrm{MSE}}_{{\mathrm{SO}}_{2}}$ decreased from 0.16±0.05 to 0.10±0.06. This strong increase in MMI can be attributed to the fact that in the first step, the initial pressure $p_{0}$ was downscaled to a resolution of 0.3 mm and then upscaled to a resolution of 0.2 mm, whereas in the second step, $p_{0}$ was downscaled to a resolution of 0.2 mm and then upscaled to a resolution of 0.1 mm. In the first step, the local maxima are consistently averaged (blurred) more than in the second step, leading to a lower maximum increase. Looking at the results of this step, we see that endotracheal illumination, away from the detector, with detector with a pitch of 0.40 mm generally performs the best. Specifically, a curved detector, endotracheal illumination away from the detector, a pitch of 0.40 mm in combination with either 0.25 or 0.5 split, and either 700 and 800, 757 and 800, or 700 and 1064 nm performs best.

## 4 Discussion

In this paper, we have investigated the performance of PA probes for thyroid nodule imaging using a digital phantom. Previous research has highlighted the use of PAI-extracted ${\mathrm{SO}}_{2}$ parameters in thyroid nodule risk stratification.^11^^,^^12^^,^^14^^,^^15^ Therefore, we evaluated the probes on both their sensitivity and their accuracy in ${\mathrm{SO}}_{2}$ estimation.

Using the MMI as a proxy for sensitivity, a statistical analysis showed that illumination location has the largest impact, followed by detector shape and split. We found that endotracheal illumination increased sensitivity most when directed toward the detection array. When directing the illumination towards the array, one points it away from the majority of the targets, which would, at first glance, be counter-intuitive. However, due to the background’s scattering, this effect is partly compensated. We also found that curved arrays consistently outperformed linear arrays in our simulations. This is because curved detector arrays can better overcome limited-view artifacts than linear detectors.^37^ Finally, under endotracheal illumination, we found that a pitch of 0.40 mm and a minimal split (i.e., no split) scored highest in MMI. Although the former complements the curved detector shape, the absence of a split is less intuitive. However, increasing the split also increases the minimum distance between the transducer elements and the targets, thereby decreasing the PA intensity.

In assessing ${\mathrm{SO}}_{2}$ estimation accuracy, a statistical analysis showed that multiple design parameters played a significant role. Concerning illumination placement, endotracheal illumination performed better than external illumination, where endotracheal illumination, directed away from the detector array, performed the best. This is in contrast with optimizing MMI, in which endotracheal illumination directed toward the array performed the best. Next, given the external illumination, the wavelength combinations involving shorter wavelengths yielded the lowest ${\mathrm{SO}}_{2}$ error, whereas under endotracheal illumination, regardless of illumination direction, the wavelength combinations involving longer wavelengths performed best. The correlation between ${\mathrm{SO}}_{2}$ estimation accuracy and wavelength combinations can be attributed to fluence decay (where increased fluence decay prevents deep tissue penetration) and spectral colouring (where increased spectral colouring hampers accurate ${\mathrm{SO}}_{2}$ estimation). External illumination requires light to pass through skin, muscle, and fat, which will distort the spectral response, whereas endotracheal illumination circumvents this problem.

We have conducted optimization of a digital setup here, but in clinical practice, the added complexity and practical feasibility of endotracheal illumination for the patient and clinician must be balanced with its added value. As a comparison, flexible bronchoscopy can be well-tolerated by patients but often requires mild sedation.^38^ Although previous studies have explored various types of internal illumination for PAI (e.g., via the pharynx^39^ or via the gastrointestinal tract^40^), endotracheal illumination would require the clinician to optimize the placement of both the detector and the illumination source, thereby increasing examination time. One thing to note is that we only changed the illumination placement in this study and not the shape of the illumination source. However, one could flood the entire neck region with light rather than targeting a single region of the thyroid, thereby reducing the importance of illumination placement optimization. Moreover, although the American National Standards Institute (ANSI) has defined limits on the maximum permissible exposure (MPE) for skin and retinal tissue,^41^ there are no official limits for tracheal lining and its surrounding tissues. To prevent tissue damage, this should be taken into account during further development.

Several limitations are present in this research, which future work could mitigate. Furthermore, although the current noise level was empirically chosen, improved noise modeling methods, such as the one from Gröhl et al.,^42^ where the system impulse response function and empty water bath noise measurements are implemented into the simulations, would improve the realism of the simulations.

The conclusions are based on a singular reconstruction technique (time reversal), with no acoustic and optical compensation. Deconvolution was applied to minimize limited frequency response artifacts, but, e.g., fluence decay and while optimizing the MMI identifies hardware setups where the targets show strong signal, the present study did not account for wavelength-dependent fluence variations. Consequently, implementation of an accurate fluence compensation strategy may change the performance of wavelength combinations and alter the identified optimum for ${\mathrm{SO}}_{2}$ estimation. Moreover, recent advances in PAI should be examined for thyroid imaging in future work. Advances in clinical PA + US systems, such as a panoramic volumetric clinical PA + US imaging system^43^ could increase the field of view, and advances in artifact removal algorithms, such as the development of an iterative optimization algorithm,^44^ an aberration correction method,^45^ and several deep learning techniques,^46^ could decrease the effect of artifacts we encountered. Finally, although we optimized the PA properties of the device, we did not consider its US performance. Especially the pitch, which one would generally speaking increase to optimize PAI image quality, as it decreases limited view artifacts, but could lead to distorted wavefronts in US.

Although the conclusions hold for the given digital phantom, the phantom itself is a simplification of a true patient, and the so-called simulation gap is still present, which will both hamper the direct translation of these conclusions to the clinic. Future work could focus on verifying our results on more complex (digital) phantoms and on patients, where the translation from the digital to the physical domain warrants further study. Physical versions of the used digital phantoms could be created for experimental validation. This is nontrivial due to the required resolution and specific physical properties, but approximations might be made using casting and moulding or additive manufacturing techniques,^47^ and iterative approximation of the physical properties.^48^ To enhance translation to the clinic, our digital experiments should be improved (e.g., by increasing the reality of the included structures in the phantoms), as well as the limitations mentioned before should be mitigated (e.g., including transducer placement and rotation as a variable). A possible approach uses scans from 3D US, MRI, or CT, of a cohort of patients, in which their segmented shapes can be used for the creation of digital phantoms.^19^ By combining different hardware parts, measurements as done in this paper can be repeated on the physical phantoms and patients, and their digital counterparts (a subsection of) our results and conclusions can be validated.

## 5 Conclusion

Our study demonstrates that photoacoustic probe performance for thyroid nodule risk assessment can be improved through optimized illumination geometry, detector design, and wavelength selection. Joint optimization of sensitivity (MMI) and ${\mathrm{SO}}_{2}$ estimation accuracy suggests that default external illumination at 757 and 800 nm, together with a curved detector (0.5 split, 0.40 mm pitch), is favorable for PAI-based thyroid imaging. However, endotracheal illumination combined using wavelengths of 700 and 800 nm, and a curved detector (0.25 split, 0.40 mm pitch) is the most favourable from a technical perspective, but for practical reasons internal illumination might not be possible. With these recommendations, insight into improved PAI hardware for thyroid nodule risk assessment is acquired. Together with US, improved PAI could lead to better diagnosis and treatment and fewer unnecessary clinical interventions.

## 6 Appendix A: Thyroid Definition

As mentioned, the thyroid is based on the phantom as designed by Hermosilla et al.^21^ Namely, the lobes are defined using

$$(x\pm 1.50{)}^{2}+(z-2.67{)}^{2}+{\left(\frac{y-1}{2.50}\right)}^{2}\leq 1,$$ (5)

and the isthmus using

$$\left\{ \begin{array}{l} x^{2}+(z-3.35{)}^{2}\leq 1.65^{2},\\ x^{2}+(z-3.35{)}^{2}\geq 1.15^{2},\\ -({\left(\frac{\arctan(\frac{\left|x\right|}{z-3.35})\mathrm{mod}\pi }{\pi }\right)}^{2})-0.8\leq y,\\ y\leq {\left(2\cdot \frac{\arctan(\frac{\left|x\right|}{z-3.35})\;\mathrm{mod}\pi }{\pi }\right)}^{2}+1,\\ z\geq 2.95 \end{array}\right.$$ (6)

with all units in cm.

## 7 Appendix B: Quantification of Design Parameter Influence

**Fig. 5:**
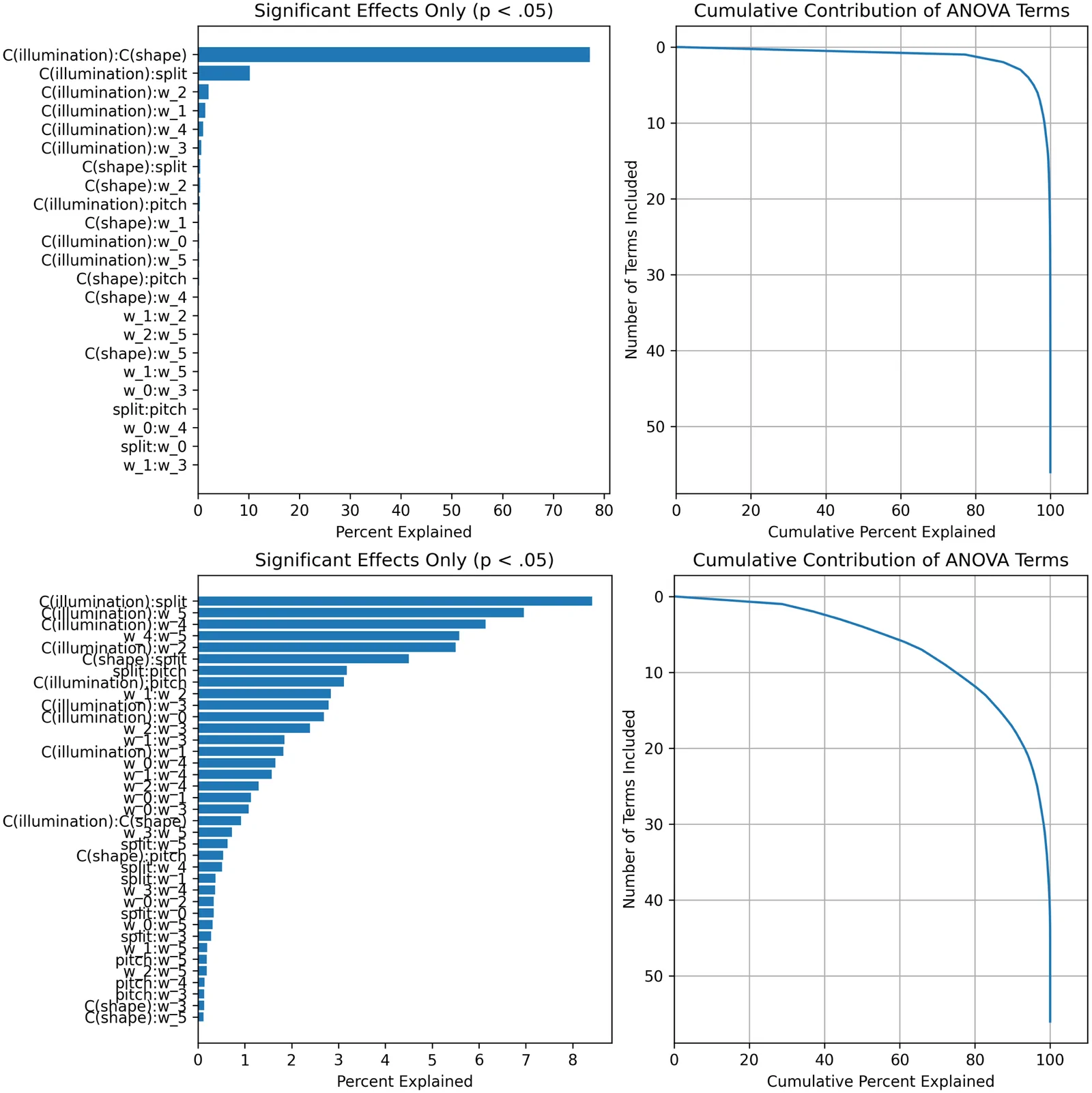
ANOVA analysis of step 1 results: MMI (top) and ${\mathrm{MSE}}_{{\mathrm{SO}}_{2}}$ (bottom).

The results of a full-factorial ANOVA between the design parameters and MMI, and between the design parameters and ${\mathrm{MSE}}_{{\mathrm{SO}}_{2}}$ are shown in Fig. 5. We also highlight the effect of illumination location and shape on the MMI in Table 2, the effect of illumination location on the ${\mathrm{MSE}}_{{\mathrm{SO}}_{2}}$ in Table 3, and the effect of illumination location and optical wavelength on the ${\mathrm{MSE}}_{{\mathrm{SO}}_{2}}$ in Table 4.

**Table 2:** Quantification of effect of illumination location, and shape on MMI.

	Setting	MMI
Illumination	External (both)	0.05±0.03
	Endotracheal (both)	0.36±0.27
Illumination	Endotracheal, away	0.27±0.18
	Endotracheal, toward	0.44±0.31
Shape	Linear	0.08±0.06
	Curved	0.33±0.29

**Table 3:** Quantification of effect of illumination location on ${\mathrm{MSE}}_{{\mathrm{SO}}_{2}}$.

	Setting	${\mathrm{MSE}}_{{\mathrm{SO}}_{2}}$
Illumination	External (both)	0.42±0.12
	Endotracheal (both)	0.32±0.07
Illumination	Endotracheal, toward	0.34±0.07
	Endotracheal, away	0.30±0.08

**Table 4:** Quantification of effect of illumination location and optical wavelength on ${\mathrm{MSE}}_{{\mathrm{SO}}_{2}}$.

Illumination	Wavelengths (nm)	${\mathrm{MSE}}_{{\mathrm{SO}}_{2}}$
Default	700 and 757	0.25±0.03
	700 and 800	0.25±0.02
	757 and 800	0.17±0.01
Side	700 and 800	0.48±0.02
	900 and 1000	0.48±0.02
	757 and 800	0.43±0.03
Endotracheal (both)	800 and 1064	0.27±0.05
	757 and 1064	0.25±0.05
	700 and 1064	0.24±0.05

## 8 Appendix C: Complete Results of Second Step

The results of the second step (specific but more realistic evaluation) are shown in Table 5.

**Table 5:** Result of the second step, sorted on their log10 MMI z-score minus their ${\mathrm{MSE}}_{{\mathrm{SO}}_{2}}$ z-score. Illumination location away indicates away from detector array, and toward indicates toward detector array.

MMI	${\mathrm{MSE}}_{{\mathrm{SO}}_{2}}$	Illumination location	Detector split	Detector shape	Pitch (mm)	Wavelengths (nm)
3.75	0.35	Endotracheal, away	0.5	Curved	0.40	1000 and 1064
3.35	0.10	Endotracheal, toward	0	Curved	0.35	800 and 1064
3.50	0.12	Endotracheal, toward	0	Curved	0.30	900 and 1064
3.47	0.09	Endotracheal, away	0.5	Curved	0.40	757 and 900
3.59	0.13	Endotracheal, away	0.5	Curved	0.40	900 and 1064
3.79	0.16	Endotracheal, away	0.5	Curved	0.40	900 and 1000
3.42	0.04	Endotracheal, away	0.5	Curved	0.40	757 and 1064
3.54	0.08	Endotracheal, toward	0	Curved	0.30	757 and 900
3.66	0.10	Endotracheal, toward	0	Curved	0.30	800 and 1064
3.73	0.09	Endotracheal, toward	0	Curved	0.30	700 and 900
3.54	0.04	Endotracheal, toward	0	Curved	0.30	757 and 1064
4.06	0.18	Endotracheal, away	0.5	Curved	0.40	800 and 1000
3.71	0.07	Endotracheal, away	0.5	Curved	0.40	757 and 1000
3.99	0.14	Endotracheal, away	0.5	Curved	0.35	800 and 1064
3.99	0.13	Endotracheal, away	0.5	Curved	0.40	800 and 1064
3.72	0.04	Endotracheal, toward	0	Curved	0.30	700 and 1064
3.79	0.03	Endotracheal, away	0.5	Curved	0.35	700 and 1064
4.01	0.09	Endotracheal, away	0.5	Curved	0.35	757 and 900
3.90	0.05	Endotracheal, away	0.5	Curved	0.35	757 and 1064
4.29	0.16	Endotracheal, away	0.5	Curved	0.40	700 and 900
3.89	0.05	Endotracheal, away	0.5	Curved	0.40	757 and 800
3.91	0.04	Endotracheal, away	0.5	Curved	0.35	700 and 800
4.10	0.08	Endotracheal, away	0.5	Curved	0.40	700 and 757
4.10	0.08	Endotracheal, away	0.25	Curved	0.40	757 and 1064
4.03	0.06	Endotracheal, away	0.5	Curved	0.35	757 and 800
4.03	0.05	Endotracheal, away	0.5	Curved	0.40	700 and 1064
4.31	0.09	Endotracheal, away	0.5	Curved	0.40	700 and 1000
4.44	0.08	Endotracheal, away	0.5	Curved	0.40	700 and 800
4.36	0.06	Endotracheal, away	0.25	Curved	0.40	757 and 800
4.41	0.06	Endotracheal, away	0.25	Curved	0.40	700 and 800

## Data Availability

The code used by this study is published at https://gitlab.com/MTRietberg/optimal-pai-hardware.
